# Quality of systematic reviews on timing of complementary feeding for early childhood allergy prevention

**DOI:** 10.1186/s12874-023-01899-4

**Published:** 2023-04-04

**Authors:** Uwe Matterne, Melissa A. Theurich, Simone Pröbstl, Dawid Pieper, Jiancong Wang, Anna Xu, Christian Apfelbacher

**Affiliations:** 1 Institute of Social Medicine and Health Systems Research, Medical Faculty, Otto von Guericke University, Leipziger Str. 44, 39120 Magdeburg, Germany; 2 Faculty of Health Sciences Brandenburg, Brandenburg Medical School (Theodor Fontane), Institute for Health Services and Health Systems Research, Rüdersdorf, Germany; 3 Center for Health Services Research, Brandenburg Medical School (Theodor Fontane), Rüdersdorf, Germany; 4 University of Oxford, Oxford, UK

**Keywords:** Allergy prevention, Complementary feeding, Methodological quality, Risk of bias, Systematic review

## Abstract

**Background:**

Only rigorously prepared analyses can provide the highest level of evidence to inform decision-making. Several recent systematic reviews (SRs) examined the hypothesis that the early introduction of specific allergenic complementary foods (CFs) to infants may lead to a lower incidence of one or more allergic outcomes. However, the methodological rigour and quality of reporting of SRs in this area has not yet been systematically evaluated.

**Methods:**

We comprehensively searched PubMed, Medline (Ovid), and Web of Science Core Collection on 13th January 2022, using a pre-specified and tested search syntax for SRs with RCT evidence on the early introduction of allergenic CFs as a means for allergy prevention in infants and children. We examined the quality and risk of bias (RoB) using AMSTAR-2 and ROBIS tools, examined adherence to the Preferred Reporting Items for SRs and Meta-Analyses (PRISMA), and checked whether certainty of the evidence was assessed.

**Results:**

Twelve SRs were included. Application of both tools resulted in similar overall judgements in terms of direction and extent for nine of the 12 SRs. Nine SRs were found to be of critically low to low quality according to AMSTAR-2 and to be at high RoB according to ROBIS. One SR received a moderate quality rating (AMSTAR-2) and high RoB rating (ROBIS). However, for two SRs, judgements between AMSTAR-2 and ROBIS were at stark variance. Only two SRs fully adhered to the PRISMA checklist. Six SRs evaluated the certainty of the body of RCT evidence. Several SRs failed to consider unpublished studies either by an explicit a priori exclusion or by inadequate search strategies.

**Conclusions:**

Well-conducted SRs are important for decision-making and informing guideline development, the quality of their methodology should therefore be considered. The methodological rigour and the reporting quality of SRs on the timing of CF for allergy prevention must be improved.

**Registration:**

https://osf.io/7cs4b.

**Supplementary Information:**

The online version contains supplementary material available at 10.1186/s12874-023-01899-4.

## Background

Allergy in children is common [1]. *Early childhood allergy prevention* (ECAP) is increasingly recognised as a potential strategy to combat the high incidence of allergic conditions [2, 3]. Recent advances have led to a shift from allergen avoidance to the early induction of tolerance paradigm as a means to prevent allergy [2, 3].

Evidence from randomised controlled trials (RCTs) on the timing of complementary foods (CF) for ECAP has emerged over the past decade [4]. Complementary feeding is the provision of foods and fluids to infants and young children, alongside breast-milk or infant formula when the latter become insufficient to meet the infants’ nutritional needs [5]. Recommendations on the optimal timing for CF with regards to ECAP have traditionally revolved around the avoidance or delayed introduction of potentially allergenic foods, particularly for at risk children [6].

This has been challenged by trials examining the hypothesis that the earlier introduction of egg, peanut, or combinations of allergenic foods may lead to a lower incidence of one or more allergic outcomes [7–13]. Several SRs and meta- analyses have synthesised both RCTs and non-randomised studies of interventions (NRSIs), which evaluated the relationship between the timing of the introduction of complementary foods and the development of one or more allergic outcomes [14–24].

Besides the general rapid increase of SRs over the years there are now indications that the number of published SRs may exceed the number of RCTs on the same topic with often substantial primary study overlap across SRs [25, 26]. Synthesised evidence, for instance in the form of high quality SRs is important. SRs provide summaries of the accumulated evidence accounting for risk of bias (RoB) and assess the certainty of the evidence, which in turn aids translation into clinical practice. It has been shown, however, that the methodological quality of SRs examining the same primary studies varies, [25, 27] which may lead to conflicting and misleading evidence syntheses, impeding the translation of knowledge to practice [25]. Consequently, substantial efforts have been made to develop tools that assist in the systematic assessment of the methodological quality of SRs and RoB in SRs. Investigations into the methodological quality of SRs on, for instance, the efficacy of probiotics have shown that 77% of the analysed SRs were rated as critically low according to the ‘Measurement Tool to Assess Systematic Reviews’ (AMSTAR-2 [28]) [29].

AMSTAR-2 [28] was developed for an assessment of the methodological quality of SRs and ROBIS [30] is an instrument to assess RoB in SRs. Methodological quality and RoB are conceptually related but still distinctly different in major aspects. Methodological quality can be understood as the methodological soundness with which a study has been conducted and RoB as whether non-implementation of sound methods may have given rise to biased results [31].

Some research has been carried out to compare these instruments with each other [32–34]. Pieper et al. [32] examined 30 SRs using both the AMSTAR-2 and ROBIS instruments, including both randomised and non-randomised studies. They found a high rate of concordance in the overall ratings of the two tools. Minor differences were attributed to AMSTAR-2 offering less scope for interpretation of variation compared to ROBIS. Perry et al. compared AMSTAR-2 and ROBIS assessments for 31 SRs concluding that SRs that included a meta-analysis were more easily rated with ROBIS while SRs without a quantitative synthesis were more easily assessed by AMSTAR-2 [33]. Another study found that 70% of the items in AMSTAR-2 and ROBIS related to same or similar methodological constructs. While inter-rater reliability was moderate to perfect for these constructs each instrument addresses unique methodological constructs as well. For instance, ROBIS addresses restrictions within eligibility criteria while AMSTAR-2 includes an assessment of the selection of study designs for inclusion and reporting on excluded studies with justification. AMSTAR – 2 addresses source of funding and reviewers’ conflict of interest while ROBIS addresses the reduction of risk of error in risk of bias assessment, completeness of data extraction or adherence to a predefined analysis plan [35].

The aim of this investigation was to examine the methodological quality, risk of bias (RoB), and reporting quality of SRs that synthesised interventional studies on the effects of earlier versus later introduction of CF on the incidence of allergy/allergic disease in infants and children. We also aimed to contrast the conclusions based on either AMSTAR-2 or ROBIS.

## Methods

The present investigation is embedded in a prospectively registered systematic review (PROSPERO (CRD42021240160)) and was registered at OSF [36]. We aimed to appraise and compare the quality of existing SRs on this subject using AMSTAR-2 and ROBIS tools and to assess whether SR reporting adhered to the Preferred Reporting Items for Systematic Reviews and Meta-Analyses (PRISMA) 2009 checklist [37]. No statistical analyses were planned prior to the commencement of the study.

### Search strategy

A comprehensive search of PubMed, Medline (Ovid), Web of Science Core Collection was conducted on 13th January 2022 using a pre-specified and tested search syntax (Appendix 1). References of included SRs were hand-searched for potentially relevant SRs. The search strategy was restricted to publications from 2010 to January 13th, 2022, because of the recency of the ‘induction of tolerance’ paradigm shift. If a SR was commissioned by an agency, we also looked for ‘unpublished’ full reports of the same review for further information. The PROSPERO database was searched for registered titles of SRs. In addition, conference abstracts from the European Academy of Allergy and Clinical Immunology (EAACI) congresses 2010 to 2021 were searched for relevant SRs.

### Eligibility criteria

SRs were eligible for inclusion if they included at least one RCT dealing with the earlier (before 6 completed months of age) versus later introduction of any CF into the diet of full-term (breastfed or formula-fed) infants. Study populations could consist of infants at heightened risk (atopic disposition) or normal risk or both, as long as separate outcomes for groups were available. SRs reporting on at least one allergic outcome (eczema, asthma, allergic rhinitis, any or specific food allergy, and/or sensitisations) were included. While the intervention was to be applied during infancy, outcome assessment could take place in infancy or childhood. When updated versions of the same SR were available, only the most recent version was included unless relevant details were only available from earlier versions.

SRs summarising only studies that had declared the complete avoidance or delayed introduction of CF as the only type of intervention irrespective of study design were excluded. SRs summarising only findings from NRSI were not eligible for inclusion.

To be included, SRs had to report at least one of the following: a research question pertaining to CF and allergy prevention, a systematic literature search in bibliographic databases, a RoB assessment of the included studies and quantitative or qualitative evidence synthesis. These criteria were derived from the definition that an SR ‘attempts to identify, appraise and synthesize all the empirical evidence that meets pre-specified eligibility criteria to answer a specific research question’ [38]. No language restrictions were imposed.

### Study selection

Based on the above eligibility criteria, three authors (UM, JW, MT) independently screened titles and abstracts in sequence. Full texts of the shortlisted references were obtained and reviewed by the same authors for eligibility. The list of excluded studies after the full-text review and the reasons for exclusions can be found in Appendix 2.

### Methodological assessment

Methodological quality assessment of each included SR was based on the review as a whole, i.e. it also considered how NRSI were incorporated into the respective SR. The Measurement Tool to Assess Systematic Reviews (AMSTAR-2) [28] was used to evaluate the methodological quality of each individual SR. AMSTAR-2 was developed for the assessment of the methodological quality of SRs that may include RCTs and/or NRSIs. It comprises 16 items, seven of which are considered critical domains and the remainder are considered non-critical weaknesses. Using this, an overall judgement can be made (high, moderate, low, or very low). AMSTAR-2 is accompanied by a guidance document specifying how the overall judgements are to be made.

RoB was evaluated by the ‘Risk of Bias in Systematic Reviews’ (ROBIS) tool [30]. It assesses the extent of bias across the four domains, study eligibility criteria, identification and selection of studies, data collection and study appraisal, data synthesis and findings. It provides an overall RoB judgement (low, high, unclear). The ROBIS tool is accompanied by a guidance document specifying how the overall judgements are to be made. In contrast to AMSTAR-2, concerns observed in the four domains of the ROBIS tool can be overcome if authors show awareness of these concerns and acknowledge them in their limitations. All articles were evaluated by pairs of reviewers independently; thus, each article was assessed by two reviewers in duplicate (UM, MT, JW). Disagreements were discussed and resolved with another reviewer (CA).

Not all SRs include a standardised evaluation of the quality of the whole body of the underlying evidence. However, the Methodological Expectations of Cochrane Intervention Reviews (MECIR) standards [39] stipulate the mandatory evaluation of the quality of the body of evidence by, for instance, GRADE (Grading of Recommendations Assessment, Development and Evaluation (GRADE)) [40]. Neither AMSTAR-2 nor ROBIS contain items assessing whether an evaluation of the certainty of the body of evidence has taken place in the respective SR, but we did assess whether the included SRs had carried out an assessment of the certainty of the evidence by GRADE or by another instrument deemed appropriate. In addition, we documented how the RoB assessment of the primary studies was undertaken in each respective SR.

### Reporting quality

We also assessed whether adherence to the PRISMA 2009 checklist items [37] (reporting completeness or quality of reporting) was pre-specified and whether authors satisfied all items on the PRISMA 2009 checklist (yes/no). The PRISMA statement is comprised of a 27-item checklist and a four-phase flow diagram. The checklist includes items regarded essential for transparent reporting of a SR [41] and is considered to be compatible with MECIR [39]. An update of the PRISMA statement and checklist was published in 2021 [42] replacing the 2009 version, hence we examined adherence to the 2009 version.

## Results

**Fig. 1:**
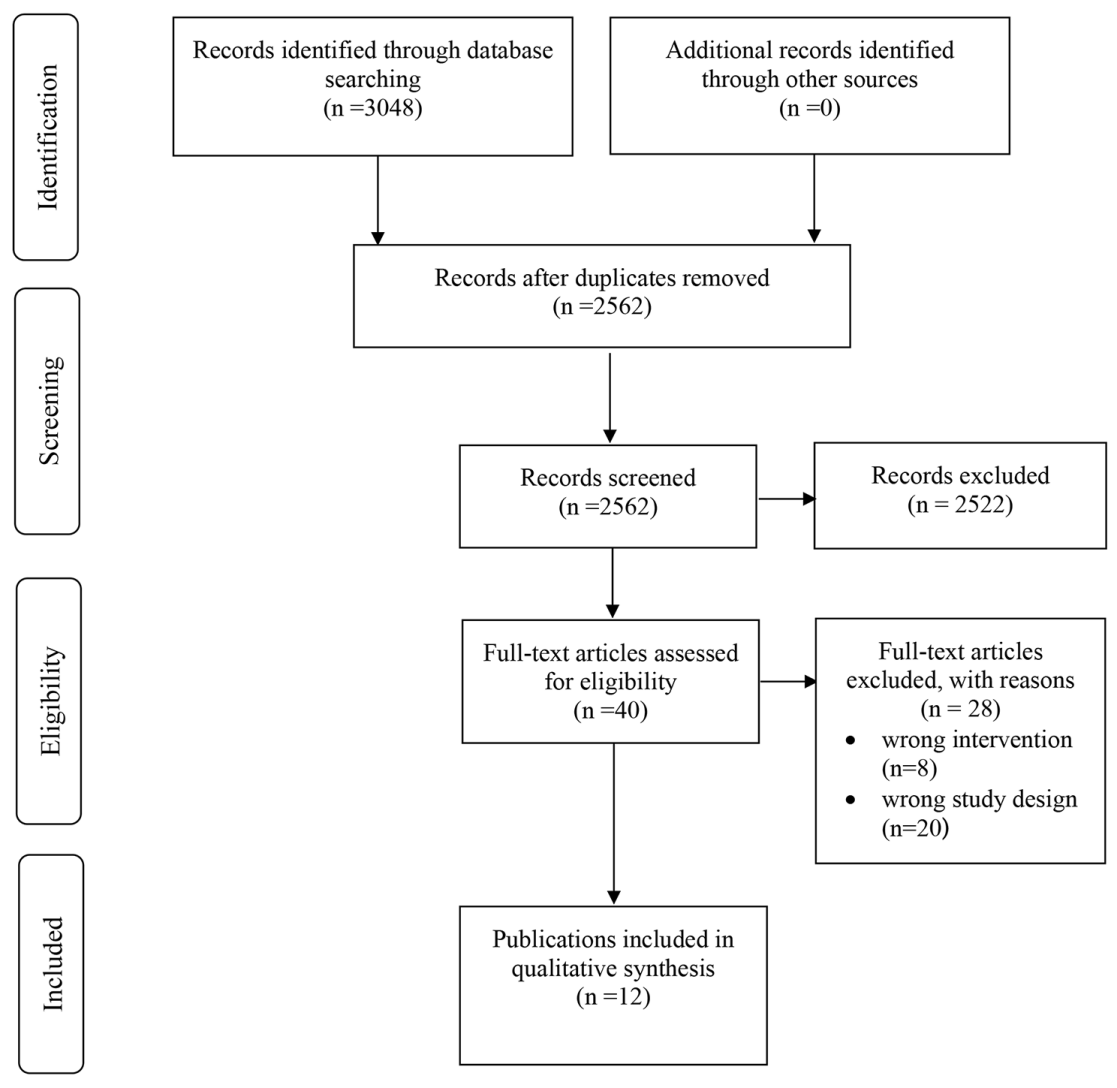
PRISMA diagram showing study selection

The searches identified 3048 potential articles. After merging and deduplication, titles and abstracts of 2562 articles were examined for eligibility and 2522 articles excluded. The remainder (n = 40) was examined based on their full texts. None of the screened conference abstracts met the inclusion criteria. Finally, 12 articles [14–24, 43] were identified as eligible for the purpose of the present investigation (Fig. 1). A list of the excluded studies and the reasons for exclusion are given in Fig. 1 and Appendix 2.

The characteristics of the 12 eligible SRs are summarised in Table 1. Ten of the 12 included SRs examined the effects of more than one CF on the incidence of allergy while two looked at the effects of a single CF. Outcomes varied from incidence of one specific food allergy (FA) to several outcomes encompassing incidence of FA and/or the atopic diseases eczema, allergic rhinitis and asthma. Three SRs included only RCTs and nine included NRSIs in addition to RCTs.

**Table 1:** Summary characteristics of included systematic reviews and their methodological assessments

Author (Year)	PICO/PECO	RCTs of interest (n)	NRSIs included	CompletePRISMA checklist adherence^†^	AMSTAR-2	ROBIS
Al-Saud (2018)	**Population**: Infants **Intervention/Exposure**: Early (as early as 3 months of age) introduction of egg to the diet **Comparator**: Exclusive breastfeeding until 6 months of age or placebo **Outcome**: Primary: Egg allergy diagnosed by oral food challenge; Secondary: food sensitization (presence of allergen specific IgE) confirmed by a positive allergy skin prick test or a positive ImmunoCAP, diagnosis of asthma, allergic rhinitis, eczema, anaphylaxis	6	No	No	Critically low quality	High RoB
Burgess (2019)	**Population**: Humans from general and high-risk populations (not specified but inferred: children 0–3 years) **Intervention/Exposure**: Timing of introduction to complementary solid food or specific allergenic foods **Comparator**: Delayed or no introduction (not explicitly stated, protocol: any comp. with infants not introduced at that age) **Outcome**: Food sensitization determined by skin prick test or food-specific IgE, food allergy determined by food challenge or diagnosed by a physician	8	Yes	No	Critically low quality	High RoB
Chmielewska (2017)	**Population**: Infants at population risk or increased risk of developing a wheat allergy **Intervention/Exposure**: Consumption of wheat- or gluten-containing products of any type (cereals, flour or any other food items containing gluten) **Comparator**: Placebo or no intervention/ no exposure **Outcome**: Incidence of wheat allergy or sensitization (increased wheat-specific IgE and/or positive skin-prick test)	1	Yes	No	Moderate quality	High RoB
Dai (2021)	**Population**: Infants at population risk and increased risk of developing allergy **Intervention/Exposure**: Late consumption of any complementary foods (after 6 months of age) **Comparator**: Early consumption of any complementary foods (before 6 months of age**)** **Outcome**: Allergic diseases (egg allergy, peanut allergy, cow’s milk protein allergy)	8	No	No	Critically low quality	High RoB
De Silva (2020)	**Population**: Infants (up to 1 year old), children (13 months to 17 years), and/or adults (18 + years) with or without an increased risk for developing allergic disease and with or without any sensitization or atopic manifestations **Intervention/Exposure**: Any intervention to prevent the development of new cases of immediate-onset food allergy (protocol: includes dietary initiatives and skin barrier initiatives) **Comparator**: Any independent, concurrently sampled group(s) with or without a placebo, intervention, or combination of interventions **Outcome**: New cases of immediate-onset food allergy, defined as a reproducible adverse response to a food protein within hours caused by an immunologic reaction	6	No	No	High quality	High RoB
EFSA (2019)	**Population**: All population groups, males and females: generally healthy term infants, preterm infants, infants not older than 12 months of age at introduction of CFs **Intervention/Exposure**: Timing of introduction to CFs **Comparator**: Group alike in terms of the type of initial feeding (breast milk or breast-milk substitutes) and the only important difference being the time at which CF is introduced **Outcome**: Allergy (+ overweight and obesity, DM type 1 and 2, risk factors of CVD, coeliac disease, dental health, renal function, gastrointestinal infections, respiratory tract infections etc.)	7	Yes	No	Low quality	High RoB
Ierodiakonou (2016)	**Population**: Infants between birth and the end of their 12th post-partum month **Intervention/Exposure**: Timing of introduction of allergenic food: cow’s milk, egg, fish, crustacean shellfish, tree nuts, wheat, peanuts and soybeans **Comparator**: Delayed or standard introduction of allergenic foods **Outcome**: Asthma/ wheeze, eczema, allergic rhinitis, food allergy (a reproducible hypersensitivity reaction to a food), allergic sensitization (the presence of specific IgE to an allergen), type 1 DM, celiac disease, IBD, juvenile rheumatoid arthritis, psoriasis, and vitiligo	8	Yes	Yes	Low quality	Low RoB
Larson (2017)	**Population**: Infants **Intervention/Exposure**: Delaying introduction of potentially allergenic foods until after 12 months of age **Comparator**: Introduction of these foods prior to 12 months **Outcome**: Development of food allergies	2	Yes	No	Critically low quality	High RoB
Obbagy (2019)	**Population**: Age at intervention or exposure: infants (0–12 mo), toddlers (12–24 mo); generally healthy infants fed human milk, infant formula, or both, examined through age 18 years **Intervention/Exposure**: Timing of introduction of CFs and beverages (CFBs), types and amounts of CFBs Different timing of introduction of CFBs, different types and amounts of CFBs **Outcome**: Incidence, prevalence, and/or severity of food allergy, atopic dermatitis/ eczema, asthma, and/or allergic rhinitis	7	Yes	No	Low quality	High RoB
Smith (2016)	**Population**: Healthy breastfeeding full-term (37–42 months of gestation) infants up to the age of six months, or mothers of these infants **Intervention/Exposure**: Non-exclusive breastfeeding infants (artificial milk, glucose, water, foods) **Comparator**: Exclusive breastfeeding infants **Outcome**: Primary: duration of breastfeeding, incidence of infant morbidity (e.g. asthma, eczema, GI infection), infant mortality, physiological jaundice; Secondary: weight, growth, development, duration of hospital stays, confidence in breastfeeding, maximum serum bilirubin levels, phototherapy	2	Yes	No	Low quality	High RoB
Waidyatillake (2018)	**Population**: Infants **Intervention/Exposure**: Timing of solid food introduction (allergenic or non-allergenic) **Comparator**: non-exposed group (e.g. infants not introduced to solid food by a certain age), if not available comparison depending on the age at introduction **Outcome**: Diagnosis of eczema	2	Yes	No	Critically low quality	High RoB
Yuan (2020)	**Population**: Not explicitly stated (infants aged 0–12 months inferred, protocol: before 12 months of age”) **Intervention/Exposure**: Timing of CM or cow’s milk formula (CMF) introduction **Comparator**: Later or no exposure to cow’s milk or cow’s milk formula **Outcome**: Development of allergic diseases, including asthma, wheeze, eczema/ atopic dermatitis, allergic rhinitis/ conjunctivitis, food allergy, and cow’s milk allergy	1	Yes	Yes	Critically low quality	High RoB

^†^adherence to all items on 2009 PRISMA checklist

The results of the methodological evaluation by AMSTAR-2 and ROBIS are given in Tables 2 and 3, and the overall judgements are displayed in Table 1.

**Table 2:** Results of AMSTAR-2 assessment of 12 included systematic reviews

		AMSTAR-2 item																									
	**1**				**2** ^†^		**3**	**4** ^†^		**5**			**6**	**7** ^†^			**8**		**9a** ^†^		**9b** ^†^		**10**	**11a** ^†^		**11b** ^†^	
**Author(Date)**																											
Al-Saud (2018)		Yes	Yes	No		PY			Yes		Yes	No			PY	PY		n.a.		No		Yes			n.a.		No
Burgess (2019)		Yes	PY	Yes		No			Yes		Yes	No			PY	Yes		PY		No		No			Yes		No
Chmielewska (2017)		Yes	PY	No		PY			Yes		Yes	Yes			No	Yes		PY		No		n.a.			n.a.		n.a.
Dai (2021)		Yes	No	No		PY			Yes		No	No			No	PY		n.a.		No		No			n.a.		Yes
De Silva (2020)		Yes	Yes	Yes		PY			Yes		Yes	Yes			Yes	Yes		Yes		Yes		n.a.			n.a.		n.a.
EFSA Panel (2019)		Yes	PY	Yes		No			No		Yes	Yes			PY	Yes		Yes		No		Yes			Yes		Yes
Ierodiakonou (2016)		Yes	Yes	Yes		Yes			Yes		Yes	No			PY	PY		PY		No		Yes			Yes		Yes
Larson (2017)		Yes	No	No		PY			No		No	No			No	No		No		No		n.a.			n.a.		n.a.
Obbagy (2019)		Yes	PY	No		No			Yes		No	Yes			PY	PY		PY		Yes		n.a.			n.a.		n.a.
Smith (2016)		Yes	Yes	No		Yes			Yes		Yes	Yes			Yes	Yes		n.a.		Yes		No			n.a.		No
Waidyatillake (2018)		Yes	PY	Yes		No			Yes		Yes	NI			PY	Yes		PY		No		No			No		No
Yuan (2020)		Yes	Yes	No		PY			Yes		Yes	No			PY	Yes		PY		No		Yes			No		Yes

Abbreviations: n.a., not applicable; PY, partial yes, NI, no information; ^†^indicates critical domain = flaw item

**Table 3:** Results of ROBIS assessments of 12 included systematic reviews

	Phase 2	Phase 3			
Review	1. STUDY ELIGIBILITY CRITERIA	2. IDENTIFICATION AND SELECTION OF STUDIES	3. DATA COLLECTION AND STUDY APPRAISAL		
Author(Date)					
Al-Saud (2018)	High risk	High risk	Low risk		
Burgess (2019)	High risk	High risk	Low risk		
Chmielewska (2017)	High risk	Low risk	Unclear risk		
Dai (2021)	High risk	Low risk	Low risk		
De Silva (2020)	Low risk	High risk	Low risk		
EFSA Panel (2019)	High risk	High risk	Low risk		
Ierodiakonou (2016)	Low risk	Low risk	Low risk		
Larson (2017)	High risk	High risk	High risk		
Obbagy (2019)	High risk	High risk	Low risk		
Smith (2016)	High risk	Low risk	Low risk		
Waidyatillake (2018)	High risk	High risk	Low risk		
Yuan (2020)	High risk	High risk	Low risk		

### SRs with disagreement between ROBIS and AMSTAR-2 assessments

Ierodiakonou et al. [16] reviewed and analysed evidence on the timing of allergenic food introduction during infancy on the risk of allergic or autoimmune disease. Their review received a “low” quality rating using AMSTAR-2 but a “low” RoB rating using ROBIS. A list of excluded studies and a justification for their exclusion was not provided, funding sources of included studies were not reported, resulting in one critical flaw and one non-critical weakness giving rise to the overall judgement of low quality (AMSTAR-2). Using ROBIS however, no concerns were raised in any of the four domains of the phase 2 assessment, hence Ierodiakonou et al. [16] had an overall judgment of low RoB.

Chmielewska et al. [22] examined whether breastfeeding duration, exclusive or any breastfeeding, and breastfeeding at the time of introducing wheat/gluten, as well as the timing of wheat/gluten introduction, influenced the risk of developing wheat allergy or wheat sensitisation. This SR received a moderate quality rating using AMSTAR-2 and a high RoB judgement using ROBIS. They provided no adequate explanation for the selection of the study designs for inclusion in the review, the included studies were not explained in adequate detail, and no information about funding sources of the included studies were provided, leading to a moderate quality judgement (no critical flaws but three non-critical weaknesses) according to AMSTAR-2. Using ROBIS, concerns arose in domain 1 (exclusion based on publication format), and in domain 4 (biases in primary studies not addressed in synthesis). In addition, we were unsure whether data collection had taken place in duplicate (domain 3). While some of these concerns were addressed in their interpretations of findings not all of them were leading to a high RoB rating (ROBIS). Thus, concerns could not be overcome, resulting in an overall high RoB rating.

De Silva et al.’s SR [24] had a broad scope, aiming to assess the effectiveness of any approach for preventing the development of immediate-onset/IgE-mediated food allergy in infants, children, and adults, compared to any other intervention or placebo. The AMSTAR-2 assessment raised no concerns, hence a high quality judgement was given. However, using ROBIS concerns arose in domain 2 (unpublished studies not sought) and domain 4 (not all predefined analyses reported, or departures explained, i.e. giving rise to selective reporting bias). As these concerns were not addressed in their interpretation of findings, it was rated as being at overall high RoB.

### SRs with agreement between ROBIS and AMSTAR-2 assessments

Al-Saud et al. [14] examined the effect of early egg introduction on egg allergy. The SR was judged as critically low quality (AMSTAR-2), and to be at high RoB (ROBIS). No list of excluded studies and no information on the role of funding of included studies was provided. There was also no indication of an instrument, such as GRADE, to assist in the interpretation of the quality of included evidence.

We noted deviations from the published trial protocol (CRD42017051345) on the stated outcome of interest (egg allergy confirmed by oral food challenge) [44] through the inclusion of a trial whose outcome was allergic sensitisation to egg [10] but not egg allergy. Another deviation was the inclusion of the Natsume et al. trial [13] in which infants in the intervention group consumed egg between 6 and 9 months of age. This was highlighted in a letter to the editor [45] in 2018. Al-Saud et al. responded to these criticisms [46] stating that enrolment and randomisation occurred before 6 months of age. However, we also consider inclusion of the Natsume et al. trial [13] to be a deviation from the SR protocol [44]. Further, we do not consider introduction of egg between 6 and 9 months of life to be an “early introduction,” in the context of ECAP.

Burgess et al. [23] aimed to synthesise the literature on the association between age at introduction of complementary solids, excluding milk products, and food allergy and sensitisation. The SR was classified as critically low quality by AMSTAR-2, and to be at high RoB, based on the ROBIS assessment.

AMSTAR-2 concerns pertained to the quality of the search, non-transparency of excluded studies, addressing heterogeneity insufficiently and an unsatisfactory discussion of the potential of publication bias. ROBIS attested to problems in study eligibility specification and in identification and selection of studies that were not addressed in the interpretation.

Dai et al. [43] synthesized literature from eight RCTs on the relationship between the timing of CF (eggs, eggs powders, peanuts and infant formula) and the occurrence of allergic diseases (egg allergy, peanut allergy, milk allergy) in infants with and without allergic predisposition. It was rated as being at high RoB by the ROBIS assessment because no study protocol or registration was identified. The AMSTAR-2 assessment revealed that no study protocol, no list of excluded studies with justifications for exclusion, and no adequate investigation of publication bias or discussion of its likely impact on the results of the review, were provided.

The SR conducted by the EFSA Panel on Nutrition, Novel Foods and Food Allergens (NDA) (EFSA) [15], aimed to assess and summarise the scientific evidence on: (1) any developmental factors relevant for the introduction of CFs, (2) any adverse health effects associated with the introduction of CFs before 6 months of age, and (3) any benefits associated with the introduction of CFs before 6 months of age. The AMSTAR- 2 assessment revealed problems with the search strategy, that sources of funding in included studies were not reported, and that conflict of interest and funding of authors was not transparently declared in the paper. Use of ROBIS demonstrated problems in study eligibility specification, in identification and selection of studies, and in synthesis and findings that were not addressed in the interpretation.

Larson et al. [18] published an SR in 2017 which aimed to explore the association between timing of introduction of potentially allergenic foods to infants and development of food allergies. ROBIS raised concerns in all four domains and AMSTAR-2 attested to several major flaws and non-critical weaknesses (ncw).

Obbagy et al. (2019) [17] examined the relationship between the timing of the introduction of complementary foods and beverages (CFBs), or types and amounts of CFBs consumed, and the development of food allergy, atopic dermatitis/eczema, asthma, and allergic rhinitis. They were downgraded to “low” quality according to AMSTAR-2 because of concerns with the search strategy. Using ROBIS, problems with study eligibility criteria, identification and selection of studies, synthesis and findings arose which were not sufficiently addressed in the interpretation of results.

Smith and Becker published a Cochrane SR in 2016 [19] aiming to assess the benefits and harms of additional food or fluid for full-term healthy breastfeeding infants and to examine impacts of the timing and type of additional food or fluid on allergy development (amongst other outcomes). Using AMSTAR-2 we found that study heterogeneity had not been sufficiently considered in the synthesis and interpretation of findings. ROBIS attested to problems with specification of the study eligibility criteria and the synthesis of findings.

The SR by Waidyatillake et al. [20] conducted in 2018 aimed to synthesise the literature on the association between age at introduction of complementary solids (excluding milk products) and food allergy and sensitisation. According to AMSTAR-2, problems arose with regard to the search strategy, addressing heterogeneity, and lack of consideration of the potential for publication bias. ROBIS identified shortcomings with eligibility criteria, the identification and selection of studies, and the synthesis and findings. These were not all sufficiently addressed in their interpretation.

Yuan et al. [21] published an SR in 2020 on evidence describing the effects of timing of cow milk or cow’s milk formula (CMF) introduction to the infant diet on the development of atopic diseases during childhood. A list of excluded studies and the underlying rationale for exclusion was not reported, and problems in the quantitative synthesis were detected (AMSTAR-2). ROBIS attested to problems with the study eligibility criteria, the identification and selection of studies, and the synthesis of findings. These issues were only partly addressed in their interpretation of findings.

### Certainty of the evidence and RoB assessment of included studies

Table 4 displays the results of whether and how the certainty of evidence was assessed (by GRADE or by another instrument), and how RoB was assessed. Because neither AMSTAR-2 nor ROBIS assess whether a formal evaluation of the quality of the body of available evidence explicitly took place, we tabulated the efforts undertaken in each SR to arrive at a grading of the reviewed body of evidence and the methods used to assess RoB in the included studies (Table 4). RoB and a quality of the evidence evaluation was done by acceptable means in five studies [14, 15, 17, 19, 24]. De Silva et al. [24] used all the tools recommended by Cochrane. An adequate quality of the evidence evaluation and RoB assessment of RCT evidence was accomplished by Ierodiakonou et al. [16]. However, RoB assessment for NRSIs was done by an unreported modified NICE checklist, and the reference given was for the STROBE (STrengthening the Reporting of Observational studies in Epidemiology) checklist [47]. Three SRs assessed RoB but did not evaluate the quality of the evidence [18, 21, 22]. Burgess et al. [23] and Waidyatillake et al. [20] provided no references for the tools used and only few results of their assessments. In the latter, SR results were supposed to be found in supplementary material which, however, appeared not to exist. Requests to obtain these data by the authors were unsuccessful.

**Table 4:** Type of assessment of risk of bias (RoB) and the certainty of the evidence within each of the 12 included systematic reviews

	RoB Assessment of RCT evidence	Evidence Grading (quality of the evidence)	NRSIs included?	RoB Assessment of NRSI evidence	Evidence Grading (quality of the evidence)	Notes
Author/Data						
Al-Saud (2018)	2011 Cochrane RoB tool	GRADE	No	n/a	n/a	
Burgess (2019)	“Cochrane Review quality assessment scale”	None	Yes	NOS	None	No reference for “Cochrane Review quality assessment scale” provided so not clear what was exactly done nor which version was used
Chmielewska (2017)	2008 Cochrane RoB tool	None	Yes	NOS	None	
Dai (2021)	Cochrane RoB tool	None	No	n/a	n/a	
De Silva (2020)	Cochrane ROB-2 tool	GRADE	Yes	ROBINS-I	GRADE	
EFSA Panel (2019)	Based on OHAT	Based on OHAT	Yes	Based on OHAT	Based on OHAT	
Ierodiakonou (2016)	2008 Cochrane RoB tool (modified)	GRADE	Yes	NICE (modified)	GRADE	Reference for NICE checklist was for STROBE, modifications not explained
Larson (2017)	SORT	None	Yes	SORT	None	
Obbagy (2019)	NEL Bias Assessment Tool	NESR Grading Rubric	Yes	NEL Bias Assessment Tool	NESR Grading Rubric	
Smith (2016)	2008 Cochrane RoB tool	GRADE	Yes (1 QRCT)	Yes	GRADE	
Waidyatillake (2018)	“Cochrane Review quality assessment scale”	None	Yes	NOS	None	No reference for “Cochrane Review quality assessment scale” provided so not clear what was exactly done nor which version was used
Yuan (2020)	2008 Cochrane RoB tool	None	Yes	NOS	None	

Abbreviations: GRADE, Grading of Recommendations, Assessment, Development and Evaluations [40]; NEL, Nutrition Evidence Library; NESR, Nutrition Evidence Systematic Review; NICE, National Institute for Clinical Excellence; NOS, Newcastle-Ottawa-Scale [48]; NRSI, Non-randomised studies of interventions; OHAT, US Office of Health Assessment and Translation [60]; QRCT, quasi-randomised controlled trial; ROBINS-I, Risk Of Bias In Non-​randomised Studies - of Interventions; RoB, Cochrane risk of bias tool; SORT, Strength of recommendation taxonomy [51]; STROBE, Strengthening the Reporting of Observational Studies in Epidemiology [47]

### Reporting quality

A PRISMA statement was provided in four published SRs and one protocol for an included SR; however, only two SRs complied with all items in the 2009 PRISMA checklist [37] (Tables 1 and 5). Larson et al. [18] had the lowest reporting quality, not adhering to 12 out of the 27 PRISMA items. For instance, the authors did not state whether a protocol existed and where it could be accessed, the full search strategy was not provided, and the process for selecting studies was not stated. Obbagy et al. [17] did not adhere to four items, including ‘protocol and registration’ and ‘risk of bias across studies’. Most of the other SRs did not adhere to two PRISMA items. The components that authors most frequently did not adhere to were ‘risk of bias across studies’ in the methods section.  [13, 17, 18, 20, 23], ‘risk of bias across studies’ in the results section  [17–20, 22], and ‘search’ [17–20]. All SRs complied with, for example, providing the rationale and objective in the introduction (items 3 and 4), stating the eligibility criteria applied (item 6), describing the numbers of studies screened and selected (item 17) and presenting study characteristics (item 18). Furthermore, all SRs provided a literature search flow chart or table. However, one provided it in rudimentary form only [18]. All but two [18, 43] had their SR registered at PROSPERO or otherwise reported a protocol registration or publication, respectively.

**Table 5:** Compliance with established criteria for registration and reporting of systematic reviews and meta-analyses

Author (Year)	PRISMA statement provided	PRISMA flowchart	Prospective registration at PROSPERO	Other protocol registration/publication
Al-Saud (2018)	Yes	Yes	Yes	No
Burgess (2019)	No	Yes	Yes	No
Chmielewska (2017)	No	Yes	Yes	No
Dai (2021)	No	Yes	No	No
De Silva (2020)	Yes	Yes	Yes	Yes
EFSA Panel (2019)	Yes^†^	No^§^	No	No^¶^
Ierodiakonou (2016)	Yes	Yes	Yes	No
Larson (2017)	No	Yes^e^	No	No
Obbagy (2019)	No	Yes	No	No^¶^
Smith (2016)	No^‡^	Yes	No^‡^	Yes^‡^
Waidyatillake (2018)	No	Yes	Yes	No
Yuan (2020)	Yes	Yes	Yes	No

^†^PRISMA statement provided in protocol; ^‡^Protocol published in Cochrane Library and Cochrane endorses adherence to MECIR standards; ^§^literature search flow table provided; ^¶^reference made to existence of a protocol but it could not be found; ^e^partly completed

## Discussion

We assessed and compared the methodological quality and RoB of SRs that had included at least one RCT providing evidence for the early introduction of CF on allergy incidence. A high quality AMSTAR-2 judgement might be expected to correspond with a low ROBIS judgement and vice versa. Application of both instruments resulted in similar overall judgements, both in terms of direction and extent for nine of the included 12 SRs, which were found to be of critically low to low quality according to AMSTAR-2 and to be at high risk of bias according to ROBIS.

For one SR, ratings were somewhat different with a moderate quality rating (AMSTAR-2) and high RoB rating (ROBIS) [22]. For two out of 12 SRs, we arrived at discordant overall judgements between AMSTAR-2 and ROBIS [16, 24]. However, despite their problematic rating by either AMSTAR-2 or ROBIS, in many aspects these two SRs were found to be of good quality. If de Silva et al. [24] had addressed the exclusion of unpublished studies and its relation to potential publication bias in their discussion and explored potentially missing studies as stated in the protocol they would have received a low RoB rating according to ROBIS. Ierodiakonou et al. [16] omitted to examine the sources of funding, and a list of excluded studies based on the full-text screening including reasons for exclusion was not published. The latter indicates a critical domain in AMSTAR-2, thus a “low” quality rating was given.

The methodological quality was found to be (critically) low in the majority of examined SRs. Quality was downgraded for several reasons using AMSTAR-2. All SRs specified their methods a priori apart from Larson et al. [18] and Dai et al. [43]. An adequate explanation of the study designs for inclusion (AMSTAR-2: ncw) was not provided by six SRs. A comprehensive search strategy was not conducted by four SRs [15, 17, 20, 23] indicating a major flaw. Restrictions imposed pertained to English language restrictions, insufficient consideration of unpublished studies, or non-provision of the search strategy. Study selection in duplicate was done by all but two SRs (ncw) and data extraction by all but three SRs (ncw). Six SRs failed to provide a list of excluded studies and the reasons for exclusion (major flaw). A sufficient characterisation of the included studies was not given by two SRs (ncw). RoB of individual studies was not satisfactorily assessed by one SR (major flaw). Because of validity concerns regarding the use of an appropriate RoB assessment tool, some SRs were slightly downgraded. For example, the Newcastle-Ottawa scale (NOS) [48] has been criticised for lack of reliability [49] and other issues [50]. It does not cover selective outcome reporting. Similarly, the tool SORT [51] has been criticised for being an overly simplified instrument that is not used internationally [52].

Nine of the 12 SRs did not consider funding sources for the included studies (ncw). Of the SRs having deemed meta-analyses appropriate, four SRs were afflicted with major flaws. Four SRs failed to assess the impact of individual studies with various RoB in their quantitative or qualitative data synthesis and three did not sufficiently consider this in their interpretation of the results. Several SRs paid little attention to heterogeneity among the included studies (major flaw) and four heeded insufficient attention to the potential of publication bias (major flaw).

Using ROBIS, two SRs were judged to have specified adequate eligibility criteria; all others were downgraded. Concerns regarding the identification and selection of relevant studies were raised by eight SRs. Data collection and study appraisal were found to be afflicted with major problems in one SR. Nine of 12 SRs were found to show major flaws in their synthesis and findings approach.

Examining whether the methods used in a SR correspond with the methodological standards expected by e.g. Cochrane may complement a thorough appraisal of the methodological quality of SRs. To improve reproducibility, SR authors in this field should make more concerted efforts to adhere to standard reporting guidelines e.g. PRISMA. Only two out of 12 SRs examined completely adhered to the PRISMA 2009 checklist [37], a finding which is in line with previous research. The pooled result of studies assessing a wide range of SRs’ adherence to the PRISMA statement suggest that reporting of many items is insufficient [53]. Adherence to PRISMA checklists is crucial as it ensures transparency of what was done, ensures reproducibility, and improves the quality of reporting [53].

Improvements in the methodological quality in many SRs could be made through providing justifications of inclusion and exclusion criteria. For example, many SRs (7, 58%) were marked as ‘No’ on item 3 (study design) of the AMSTAR-2 tool because authors did not provide the rationale for their selection of study designs for inclusion. This can be improved in future SR updates by providing justifications or by amending the study designs chosen with respective justifications.

While one SR and meta-analysis showed that there is no evidence of systematic bias when English-language restrictions are applied in SR and meta-analyses [54], the Cochrane Handbook for Systematic Reviews of Intervention [55] suggests that authors may introduce language bias into their results by using restrictive language criteria [55]. This potential source of bias has hence been taken up in item 1.5 in the ROBIS tool (‘Were any restrictions in eligibility criteria based on sources of information appropriate (e.g. publication status or format, language, availability of data)?’) and in item 4 in the AMSTAR-2 tool (‘Did the authors of this review use a comprehensive literature search strategy?’). In terms of language restrictions applied by the SRs, many SRs were downgraded because they used restrictive eligibility criteria such as only including studies in the English language, without giving any justification for applying this type of restriction. Several included SRs applied English language restrictions. Future updates could expand the SR search strategies to include non-English studies; or justify not doing so.

### Empirical relationship between AMSTAR-2 and ROBIS

A previous study applied both AMSTAR-2 and ROBIS to the same body of SRs and reported a good rate of agreement between the two tools’ overall assessment results [32]. They calculated Gwet’s AC_1_ as a measure of correspondence between fully and partially overlapping AMSTAR-2 and ROBIS items and found them to range between 0.38 and 0.84 with a median of 0.69. Jaca et al. [56] recommend use of either AMSTAR (not AMSTAR-2) or ROBIS based on PCA analysis of 57 SRs. However, they found 32% to be “high”, 60% “moderate” and 9% “low” quality using AMSTAR. Using ROBIS, they judged 74% at “low”, 14% at “unclear” and 12% at “high” RoB. Perry et al. [57] applied both AMSTAR-2 and ROBIS to 16 SRs. Two studies were judged to be at “low” RoB according to ROBIS and “low” quality according to AMSTAR-2.

These findings suggest that the application of the two tools appears to lead to corresponding overall judgements in a substantial amount of SRs, but in a non-negligible number of SRs it may lead to opposing judgements.

Another study found that a large percentage of SRs judged at “high” quality using AMSTAR were found to be at “high” RoB using ROBIS [58].

From our experience with AMSTAR-2 and ROBIS, we cannot unreservedly recommend use of either tool alone but recommend complementary use. For example, in this study, confidence in the quality of a particular SR would dramatically vary in the case of the SRs conducted by Ierodiakonou et al. [16] and de Silva et al. [24] based on the tool used. Users of assessment tools should be aware that assessments of methodological quality or RoB show slight differences in their conceptual approach.

Besides the complementary assessments, we agree with Hennessy and Johnson [59] that additional information is sought to aid in the interpretation of the results of the two tools, such as an elaborate analysis of primary study overlap based on their suggested five-stage process. Further additional information could also refer to a more detailed analysis of reporting quality (e.g. adherence to protocol and PRISMA checklist) and whether SR authors assessed the certainty of the evidence.

### Strengths and limitations

Other authors may have arrived at slightly different AMSTAR-2 and ROBIS ratings because several items are subject to interpretation. All assessments were, however, done in duplicate and disagreements resolved by arbitration with a third reviewer. We acknowledge that our sample of N = 12 SR was rather small. A larger sample might have yielded similar or dissimilar findings particularly regarding the concordance between AMSTAR-2 and ROBIS. However, we dealt with a total SR sample in complementary feeding for early childhood allergy prevention.

## Conclusions

Based on our assessments using the AMSTAR-2 tool, only a single SR received a rating of high quality, hence we conclude that the methodological rigour of SRs in this area is poor. Based on our assessments using the ROBIS tool, only one SR received an overall risk of bias judgment of low. Therefore, we conclude that the risk of bias for SRs on this topic is high. We also conclude that the methodological rigour and the risk of bias for SRs on the topic of CF interventions for childhood allergy prevention should be improved. Due to the relatively large number of SRs identified on this specific subject (n = 12), it is recommended, where possible, to address the concerns raised on ROB and SR methodology in order to improve the quality of existing SRs, for example, through periodic updates, rather than conducting additional SRs on the subject. Users of assessment tools should be aware that assessments of methodological quality or RoB are conceptually different.

### Deviations from protocol

The protocol did not explicitly state what conditions a publication had to meet in order to be eligible as an SR for the purpose of the present investigation. More explicit eligibility criteria were added to the methods section.

## Electronic supplementary material

Below is the link to the electronic supplementary material.

Supplementary Material 1

## Data Availability

The datasets used and/or analysed during the current study are available from the corresponding author on reasonable request.
