# Supplementary material for: Quality of systematic reviews on timing of complementary feeding for early childhood allergy prevention
Source: BMC Med Res Methodol. 2023 Apr 4;23:80. doi: 10.1186/s12874-023-01899-4 (PMC10071735; doi:10.1186/s12874-023-01899-4)
Supplement: Supplementary file 1 — Supplementary Material 1 [file 12874_2023_1899_MOESM1_ESM.docx]

**Appendix 1 Search strategy**

**Medline (Ovid)**

(exp infant/ or Child, Preschool/ or (child or children).ti,ab,kf. or (pre-school$ or preschool$).ti,ab,kf. or Nurseries/ or (nursery or nurseries).ti,ab,kf. or exp Parents/ or (parent or parents or mother or mothers).ti,ab,kf. or (infant or infants).ti,ab,kf. or infancy.ti,ab,kf. or toddler?.ti,ab,kf. or (baby or babies).ti,ab,kf. or newborn$.ti,ab,kf. or neonat$.ti,ab,kf. or Pediatrics/ or (pediatric$ or paediatric$).ti,ab,kf. or early childhood.ti,ab,kf. or (Pregnant Women/ or Pregnancy/ or Prenatal Nutritional Physiological Phenomena/) or pregnan$.ti,ab,kf. or Prenatal Exposure Delayed Effects/ or Maternal Exposure/ or ((maternal or prenatal) adj1 exposure$).ti,ab,kf. or (fetus or fetuses or fetal or foetus or foetuses or foetal).ti,ab,kf. or Fetus/ )

AND

(exp Preventive Health Services/ or Preventive Medicine/ or "prevention control".fs. or prevent$.ti,ab,kf. or prophyla$.ti,ab,kf. or Infant Formula/ or (formula or supplement$).ti,ab,kf. or ((risk or protect$ or development or avoidance or exposure or introduction) adj6 (allerg$ or hypersensitivit$ or atopy or atopic or dermatitis or neurodermatitis or asthma)).ti,ab,kf. )

AND

(exp Hypersensitivity/ or Allergens/ or allerg$.ti,ab,kf. or hypersensitivit$.ti,ab,kf. or prick test$.ti,ab,kf. or exp asthma/ or Dyspnea/ or (asthma$ or dyspnea or wheezing).ti,ab,kf. or (difficult$ adj1 breathing).ti,ab,kf. or rhinoconjunctivitis.ti,ab,kf. or (atopic adj1 (dermatit$ or neurodermatit$ or eczema or disease)).ti,ab,kf. or Diaper Rash/ or ((infant or infantile or diaper) adj1 (rash or rashes or eczema or dermatit$)).ti,ab,kf. or Disseminated Neurodermat$.ti,ab,kf. )

AND

((systematic review) or (meta-analysis) NOT (exp animals/ not humans.sh.))

And

(2010:2021.(sa_year))

**Pubmed**

("allergie"[All Fields] OR "hypersensitivity"[MeSH Terms] OR "hypersensitivity"[All Fields] OR "allergies"[All Fields] OR "allergy"[All Fields] OR "allergy and immunology"[MeSH Terms] OR ("allergy"[All Fields] AND "immunology"[All Fields]) OR "allergy and immunology"[All Fields] OR ("eczema"[MeSH Terms] OR "eczema"[All Fields] OR "eczemas"[All Fields]))

AND

("prevent"[All Fields] OR "preventability"[All Fields] OR "preventable"[All Fields] OR "preventative"[All Fields] OR "preventatively"[All Fields] OR "preventatives"[All Fields] OR "prevented"[All Fields] OR "preventing"[All Fields] OR "prevention and control"[MeSH Subheading] OR ("prevention"[All Fields] AND "control"[All Fields]) OR "prevention and control"[All Fields] OR "prevention"[All Fields] OR "prevention s"[All Fields] OR "preventions"[All Fields] OR "preventive"[All Fields] OR "preventively"[All Fields] OR "preventives"[All Fields] OR "prevents"[All Fields] OR

"dietary supplements"[MeSH Terms] OR ("dietary"[All Fields] AND "supplements"[All Fields]) OR "dietary supplements"[All Fields] OR "supplement"[All Fields] OR "supplement s"[All Fields] OR "supplemented"[All Fields] OR "supplementing"[All Fields] OR "supplements"[All Fields] OR

"probiotic s"[All Fields] OR "probiotical"[All Fields] OR "probiotics"[MeSH Terms] OR "probiotics"[All Fields] OR "probiotic"[All Fields]) OR ("prebiotically"[All Fields] OR "prebiotics"[MeSH Terms] OR "prebiotics"[All Fields] OR "prebiotic"[All Fields])

AND

("systematic review"[Publication Type] OR "systematic reviews as topic"[MeSH Terms] OR "systematic review"[All Fields] OR "meta analysis"[Publication Type] OR "meta analysis as topic"[MeSH Terms] OR "meta analysis"[All Fields])

AND

(2000/01/01:3000/12/31[Date - Publication])

**Web of Science (core collection)**

(TS=(child*) OR TS=(infant*) OR TS=(pediatric) OR TS=(paediatric) OR TS=(babies) OR TS=(toddler*))

AND

(TS=(prevent*) OR TS=(protect*))

AND

(TS=(allerg*) OR TS=(eczema) OR TS=(dermatitis) OR TS=(atop*) OR TS=(asthma) OR TS=(sensiti*))

AND

(TS=(systematic review) OR TS=(review) OR TS=(meta-analysis))

AND

(PY=(2000-2021))

**Appendix 2 Excluded references and reasons for exclusion**

| **SR** | **Reason for exclusion** | **Note** |
| --- | --- | --- |
| Calamelli (2018) | Wrong study design | No SR |
| Chiale (2021) | Wrong study design | No SR according to eligibility criteria, preterm infants |
| De Silva (2014) | Wrong study design | No RCT on early introduction included in SR |
| Dogaru (2014) | Wrong intervention | Role of breastfeeding |
| Fanning (2021) | Wrong study design | Economic evaluation |
| Feng (2021) | Wrong study design | SR of observational studies |
| Garcia-Marcos (2013) | Wrong study design | No RCT included in SR |
| Garcia-Larsen (2018) | Wrong intervention | No RCT on early introduction included in SR |
| Güngör (2019) | Wrong intervention | Milk feeding practices |
| Halken (2004) | Wrong study design | No SR |
| Heuven (2021) | Wrong intervention | No RCT on early introduction included in SR |
| Host (2008) | Wrong study design | No SR |
| Hosseini (2017) | Wrong study design | No RCT included in SR |
| Kremmyda (2011) | Wrong intervention | No RCTs on early introduction |
| Lin (2020) | Wrong study design | No RCT on early introduction included in SR |
| Lv (2014) | Wrong study design | No RCT included in SR |
| McGowan (2014) | Wrong study design | Comment on de Silva 2014 |
| Muraro (2021) | Wrong study design | No RCT on early introduction included in SR |
| Nurmatov (2011) | Wrong study design | No RCT included in SR |
| Robison (2010) | Wrong study design | No RCT included in SR |
| Seyedrezazadeh (2014) | Wrong study design | No RCT included in SR |
| Szajewska (2011) | Wrong study design | No SR |
| Thompson (2010) | Wrong intervention | No RCT on early introduction included in SR |
| Van Brakel (2020) | Wrong intervention | No RCT on early introduction included in SR |
| West (2017) | Wrong study design | No SR |
| Xue (2021) | Wrong study design | No RCT on early introduction included in SR |
| Zhang (2020) | Wrong study design | No RCT included in SR |
| Zhang (2017) | Wrong intervention | Included RCT on maternal intervention |

*Note:* The table lists the excluded articles with at least one reason for exclusion but may not reflect all possible reasons
